# Vive la difference! Celebrating and supporting autistic psychiatrists with autistic doctors international

**DOI:** 10.1192/bjo.2021.157

**Published:** 2021-06-18

**Authors:** Sue McCowan, Sebastian C K Shaw, Mary Doherty, Bernadette Grosjean, Paula Blank, Malcolm Kinnear

**Affiliations:** 1 Associate Specialist Doctor, Old Age Psychiatry, Dorset Healthcare University NHS Foundation Trust; 2 Honorary Clinical Lecturer, Department of Medical Education, Brighton and Sussex Medical School; 3 Founder of Autistic Doctors International, Consultant Anaesthetist, Our Lady's Hospital, Navan; 4 Retired Associate Professor of Psychiatry, University of California Los Angeles; 5 Consultant Child and Adolescent Psychiatrist, UK; 6 Consultant General Adult Psychiatrist, NHS Fife, Honorary Senior Clinical Teacher, University of Dundee

## Abstract

**Aims:**

We aim to raise awareness of the existence and value of autistic doctors in psychiatry and to also signpost psychiatrists who are or suspect they might be autistic towards peer support.

**Method:**

Autism refers to a lifelong difference in how people communicate and interact with the world. These differences lead to strengths and challenges with individual profiles which include special interests, hyper-focus, and often sensory differences and anxiety. Autism has an estimated prevalence of 1-2%, which is likely an underestimate. It was noted that there was little in the way of advocacy for autistic doctors around the world. Anecdotal evidence also suggested possible issues of misunderstanding and stigmatisation of autistic doctors. As such, there was a need to tackle this to promote positive change. MD founded the group Autistic Doctors International (ADI) in 2019 to foster camaraderie, advocacy and support. ADI has flourished with 250+ members currently. In a recent member poll, 24 of 180 respondents identified themselves as psychiatrists – second only to general practice (n = 54). Several other consultant psychiatrists are known to self-identify as autistic but have not formally joined due to the fear of disclosure. The group has additionally supported multiple doctors to tackle prejudice and discrimination in the workplace / training environment. It has also brought together autistic doctors with academic interests and has generated multiple academic outputs in the form of publications, research grants and conference posters/papers regarding autism.

**Result:**

Psychiatrists, and doctors in general, are a self-selecting group for many autistic strengths such as hyper-focus, curiosity, self-motivation, a desire to study social communication, attention to detail, pattern recognition, problem solving and empathy, which, contrary to prevailing stereotypes, can be marked in autism. The increasing numbers of doctors joining ADI supports the assumption that autistic individuals are safe and effective clinicians. It is worth noting that many members are not ‘doctors in difficulty’. Those who have been able to achieve suitable accommodations, often without realising why they were needed, have flourished. Such accommodations and outcomes are in line with the neurodiversity movement, which promotes a view of autism as difference, rather than pure disability or disorder. This aims to challenge stereotypes and the tragedy narrative surrounding autism.

**Conclusion:**

Autism awareness is increasing amongst doctors but more open discussion is still needed in order to facilitate appropriate peer and workplace support. This is likely to improve mental wellbeing and resilience for autistic psychiatrists.

